# Disability Weights Estimates From India in 2018: Measurements From Community Members From Two Distinct States of India

**DOI:** 10.3389/fpubh.2022.752311

**Published:** 2022-03-22

**Authors:** Lipika Nanda, Eunice Lobo, Geetha R. Menon, Pratik Dhopte, Shuchi Sree Akhouri, Chandni Shrivastava, Roshan Ronghang, Aiswarya Anilkumar, Ambarish Dutta

**Affiliations:** ^1^Indian Institute of Public Health-Hyderabad, Public Health Foundation of India, Hyderabad, India; ^2^Indian Institute of Public Health-Bengaluru, Public Health Foundation of India, Bengaluru, India; ^3^National Institute of Medical Statistics, Indian Council of Medical Research, New Delhi, India; ^4^Health Economics and Outcome Research, Mumbai, India; ^5^Department of Concurrent Measurements and Learning, Care India, Patna, India; ^6^National Health Mission, Chhattisgarh, India; ^7^National Health Mission, Meghalaya, India; ^8^Indian Institute of Public Health-Bhubaneswar, Public Health Foundation of India, Bhubaneswar, India

**Keywords:** disability weights, global burden of diseases, health state valuation, community, India

## Abstract

**Background:**

India is undergoing a rapid demographic and epidemiologic transition. Thus demanding prioritization of diseases based on burden estimation is befitting our cultural diversity. Disability weights (DWs) by Global burden of disease (GBD) studies may not be representative. Hence, a study was conducted to estimate state-specific disability weights to capture the community health perceptions that included urban–rural settings as well as different socio-economic and literacy levels.

**Methods:**

A total of 2,055 community members (participants) from two distinct states of India, Odisha and Telangana, were interviewed to assign disability weights to the selected 14 health states based on the state burden and relevance. Each health state was described to the participants using pictorial representations of the health states and valuated using visual analog scale and card sort methods.

**Results:**

We noted that DWs in Odisha ranged from 0.32 (0.30–0.34) for upper limb fracture due to road traffic accident (least severe) to 0.90 (0.88–0.93) for breast cancer (most severe) among the 14 health states. While, in Telangana, diarrhea was considered least severe [DW = 0.22 (0.19–0.24)] and breast cancer remained most severe [DW = 0.85 (0.83–0.88)] as in Odisha. Marked difference in the DWs for other health states was also seen. Further, on comparison of community weights with GBD weights using Spearman correlation, we observed a low correlation (ρ = 0.104).

**Conclusion:**

Our study provides community-based findings that show how participants valued noncommunicable diseases higher than short-term ailments or infectious diseases. Additionally, the low correlation between GBD also suggests the need for local disability weights rather than universal acceptance. We therefore recommend that decisions in policy-making, especially for resource allocation and priority setting, need to be based not only on expert opinion but also include community in accordance with high scientific standards.

## Introduction

In the last three decades, India has been experiencing a rapid epidemiological shift owing to the increase in aging population and structural changes in disease patterns (1). Such changes imply that it is essential to prioritize the health states based on their relative burden across the country to foster efficient policy planning and thereby an effective allocation of resources. The Global burden of disease (GBD) initiative by Murray et al. in the 1990s (2) was a major stride in this regard. The study introduced disability-adjusted life years (DALYs) as a single metric measuring the disabling power of any disease (in terms of mortality and morbidity) and enabled comparison across different health conditions to support evidence-based decision making (3). Disability weights (DWs), an essential component of DALYs, reflect the relative severity of health states as a scaled measurement. Computation of these DWs is broadly a two-step procedure. Firstly, it requires the quantification of different health states through a rigorous valuation or scoring by the valuers or respondents based on their perspective and understanding of a disease condition. This is usually done using different methods for health state valuation. Secondly, the scores obtained after health state valuation are used to arrive at the DWs through multiple computational approaches.

However, even over the years, the weights derived from the various GBD studies lack representativeness of the socially and educationally vulnerable populations. Though the GBD 2010 study addressed the criticism by reestimating the DWs after a valuation that tried to incorporate the opinions of socially and culturally diverse populations, a majority of respondents included in the survey had tertiary level education at least. Subsequently, various health state valuation studies have been conducted across the globe over the past decade to establish DWs using different health state valuation methods as person trade-off (PTO), time trade-off (TTO), paired comparison (PC), standard gamble (SG), and visual analog scale (VAS). Some of these studies have been described briefly in the Table 1.

**Table 1:** List of various health state valuation studies conducted across the globe and the valuation methods used.

**S.no**	**Reference**	**Year**	**Health state description**	**Valuation method**	**Study population**
1	Murray et al. (4)	1996	DS	PTO, VAS	Medical professionals
2	Stouthard et al. (5)	1997	DS+ EQ-5D	PTO, VAS	Medical professionals
3	Jelsma et al. (6)	2000	-	VAS	General population and medical professionals
4	Baltussen et al. (7)	2002	DS	VAS	Rural population and medical professionals
5	Schwarzinger et al. (8)	2003	DS+ EQ-5D	VAS, TTO, PTO	Medical and non-medical (educated) professionals
6	Haagsma et al. (9)	2008	DS+ EQ-5D	VAS, PTO	Educated population
7	Salomon et al. (10)	2012	DS(without labels)	PC	General population (mostly educated)

^*^DS, disease specific; EQ-5D, Euro QOL 5 dimensions (functional status description); PTO, Person trade-off; TTO, Time trade-off; VAS, Visual analog scale; PC, Paired comparison.

Most of the studies listed in Table 1 were conducted on the educated population using rather challenging methods of valuation, and the perception of the lesser educated or rural or urban poor was unaccounted for. Additionally, an important gap in the literature and survey design is helping respondents and policy-makers distinguish among several factors likely affecting the disability weight assigned to a condition, such as: its severity, duration, and availability of treatment. If the description of the disease state does not include these items, respondents in different contexts will likely have contrasting impressions and generate disparate disability weights. Further, studies also suggest that health is greatly influenced by socio-cultural differences as well as geographical variations and thus guided by the perception of the people. For a country as geographico-culturally diverse as India with a large population of lesser educated and rural inhabitants, it is bound to have manifold health perceptions. However, there is a paucity of community-derived disability weights, especially in India. A relevant study was done by Mahapatra et al. (11), in a single village of ~1,000 rural participants from Andhra Pradesh in India almost two decades ago (in the year 2000) to establish community-derived disability weights. The tools and methods used require an urgent revision, refinement, and contextualization to the current societal changes and health states accounting for epidemiological and demographical transitions.

Hence, there is a vital requirement to focus on health state valuation of the general population for obtaining disability weights for health states that would be country- and state-specific that captured the community perception. Although Art and Science are entirely different from one another, they have been known to influence each other. One helps the other in creating knowledge that is distinct. However, when both are used together, the results enhance the value of the knowledge and the product. Through the use of visual analog scale method in our study, we used the opportunity to use visual graphics. Knowingly, we made use of art and science to add value to our study such that the information of the selected health states would aid the participants in better visualization and understanding. There is an urgent need to provide experts and laypersons alike tools that allow easy comprehension of health states and the means to help obtain disability weights. Thus, we conducted a study to estimate disability weights assigned by communities for various health conditions in two distinct states of India across different settings as urban and rural, as well as different socio-economic and literacy levels.

## Methods

### Health States and Description

An array of health states was selected that represented the region- and country-specific diseases and injuries. A total of 14 health states were selected based on the state burden and relevance. Three individual exercises were undertaken to shortlist health states: (i) review of literature from PubMed, Institute of Health Metrics and Evaluation (IHME) databases; (ii) consultation with medical experts (primary care and specialist providers); (iii) community exploration in urban slums and rural pockets. The selection procedure attempted to encompass various health conditions that represented the broad spectrum of diseases and injuries afflicting human population, which were also assessed by the GBD studies. Hence the health states included: communicable, nutritional diseases, such as diarrhea, tuberculosis, malaria, anemia; noncommunicable diseases including mental health, such as diabetes, quadriplegia due to stroke, oral and breast cancer, osteoarthritis, asthma, schizophrenia, depression, alcohol use disorder; and injuries, such as upper limb fracture due to road traffic accident. Detailed explanation of the process has been published earlier (12).

Each health state's descriptions were developed by means of thorough discussions with medical experts and team consensus. These descriptions included salient clinical symptoms characteristic of the given health state, along with the modified EuroQol EQ-5D+ (13, 14) instrument to further describe the health state's functional status. Six dimensions of EuroQol (“mobility,” “self -care,” “usual activities,” “pain/discomfort,” and “anxiety/depression” along with “cognition”) were used in the present study with three levels of severity in each dimension with 1 = no problem, 2 = some problem, and 3 = severe problem.

**Figure 1:**
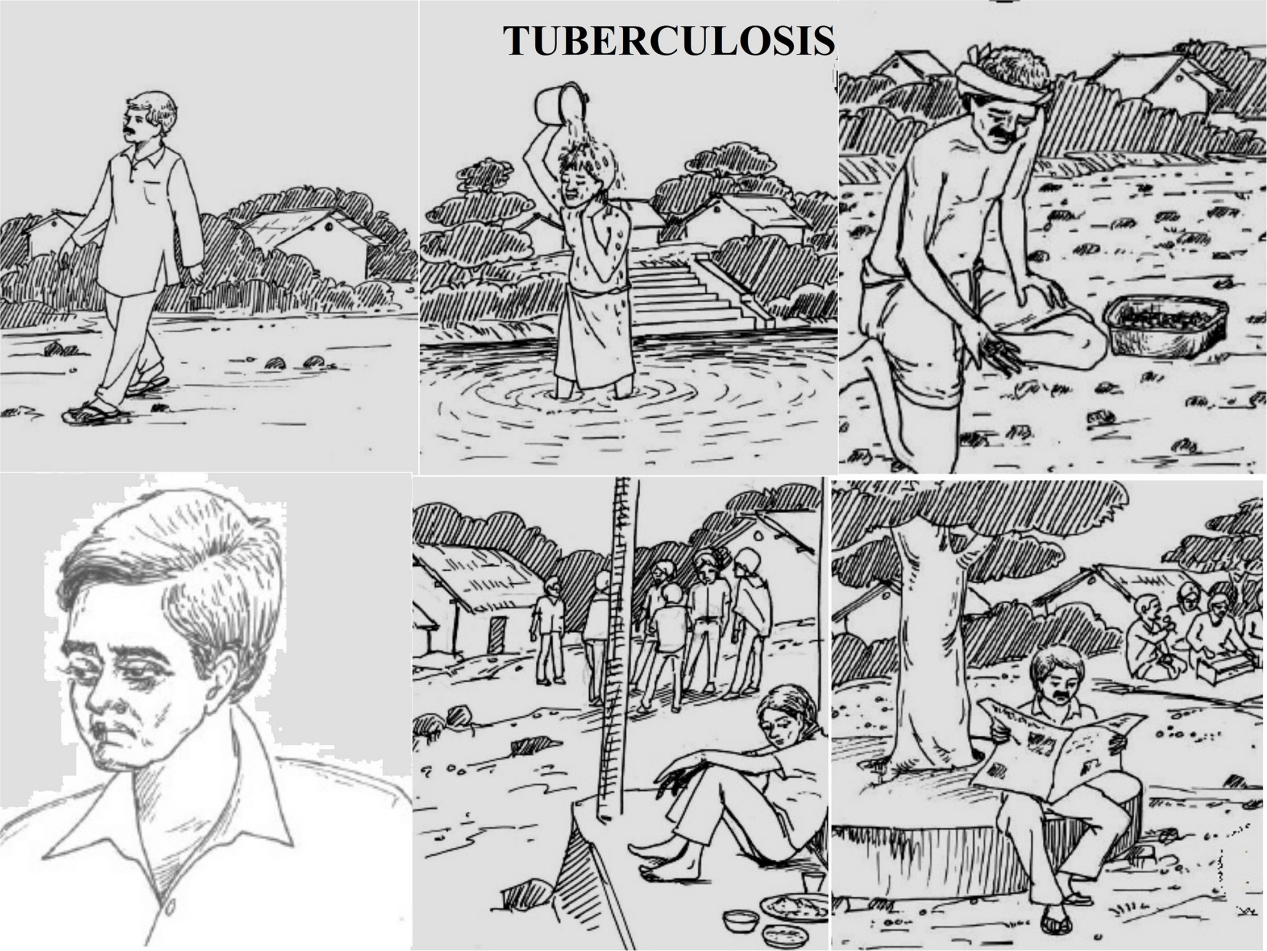
Example of health state description, tuberculosis.

For instance, the health state “tuberculosis” was described according to cardinal symptoms, prognosis, treatment along with an image of functional status describing varying levels of each dimensions (Figure 1).

### Tuberculosis

#### Clinical Description

Patient with:

- cough for more than 2 to 3 weeks (average 15 days)
- hemoptysis
- weakness
- fever
- under anti-TB treatment.

#### Functional Status Description

- No problems in walking
- No problem in washing or dressing self
- Some problem with performing usual activities (work, study, housework, or leisure)
- Some pain or discomfort
- Moderately anxious or depressed
- No cognitive impairment (concentration, memory, orientation).

### Study Setting, Design, and Sampling

**Figure 2:**
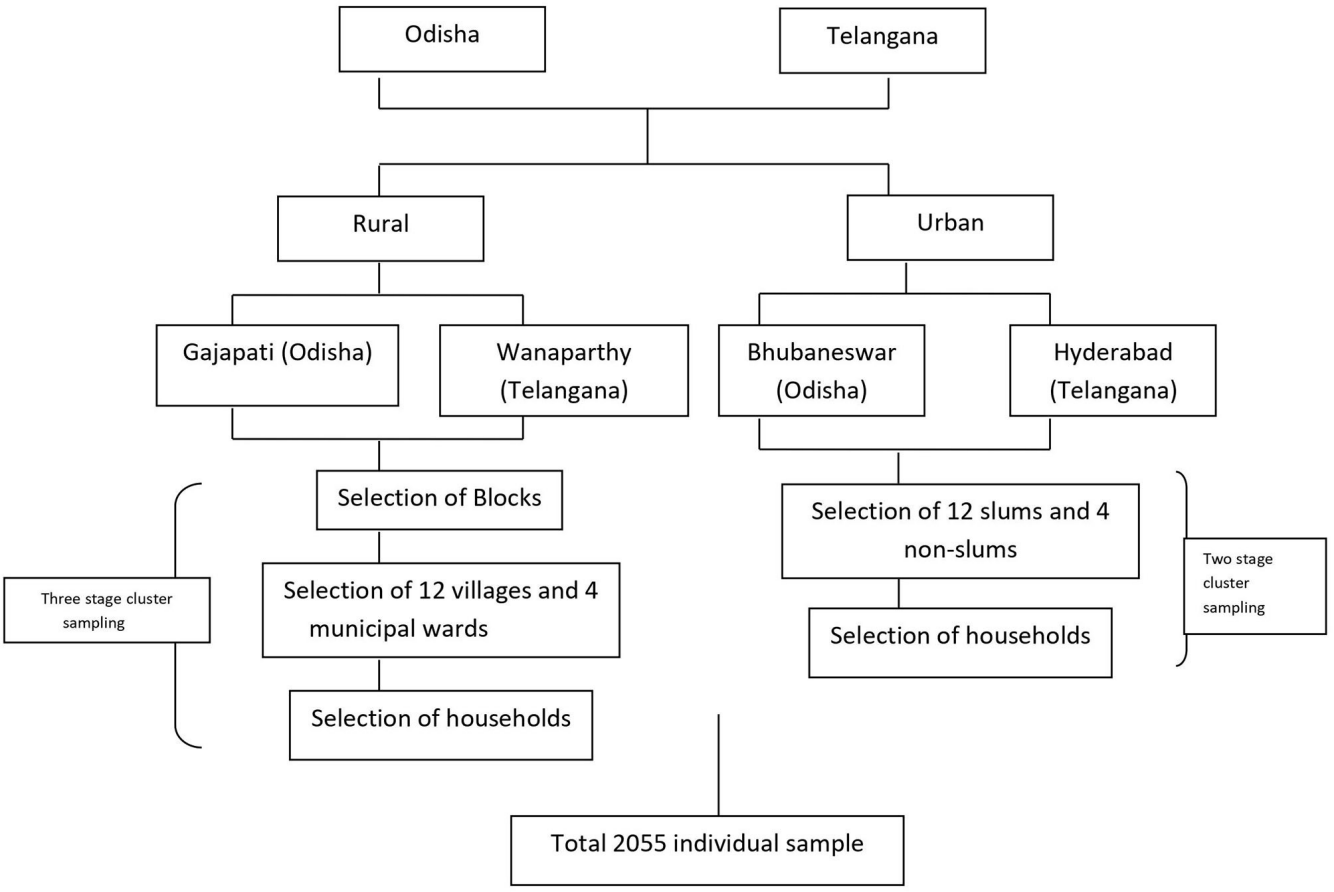
Multistage cluster sampling strategy.

The study was conducted with community members (participants) from two distinct states of India, Odisha and Telangana. The neighboring states were purposefully selected due to their cultural differences with a focus on urban–rural dissimilarities. Hence Gajapati and Wanaparthy districts for rural, and state capitals Bhubaneswar and Hyderabad, from Odisha and Telangana were chosen, respectively. To ensure representation of community members, 2,055 individuals were sampled using a multistage-stratified cluster design to ensure the probability of selection proportional to population size. Hence, a three-stage sampling technique for rural (12 villages and four wards including municipal corporations) and a two-stage for urban setting (12 slums and four nonslums) were adopted (Figure 2).

### Data Collection: Participants and Valuation Procedure

Previous studies suggest that cognitively less demanding methods as visual analog scale (VAS) (15, 16) are adept for generating scores used to calculate disability weights from individuals of varied backgrounds. Hence, we valuated the proposed health states through the visual analog scale method after a warm-up exercise using card sort or ranking of diseases. The VAS method uses a continuous graduated line segment, one end labeled as “death” and the other labeled as “perfect health” ranging from 0 to 100. It allows the user to rate a particular health state between the mentioned anchor points. The card sorting exercise further helped to strengthen the process of arriving at the final VAS scores through various iterative rounds.

The community survey was done through face-to-face interviews with consenting participants from February to May 2018 by trained public health researchers in the preferred local language of the participants (Odia and Telugu). Community members residing in the selected location, aged 18 years, and above with an acceptable level of cognitive functioning who provided their written consent were included in the study.

To reduce cognitive burden during the valuation process, every participant valued 11 health states including location- and gender-specific diseases.

The valuation process was divided into two parts:

1. After the participant valued their “own health”, individual health states were read aloud and they were asked to rank the health states in their preferred order of severity, starting with the less severe between 1 and 5, and more severe between 6 and 11.
2. The participants were then asked to rate proposed health states on a scale ranging from 0 to 100, where 0 indicates “undesirable health state” and 100 “most desirable health state”.
3. Iterations were conducted by the participant until harmonization between card sort ranking and VAS scores were acquired. “Final” scores were noted in the data sheets.

### Data Analysis

Quantitative data analysis was done using R version 3.2.2. Descriptive statistics of the socio-demographic participant profile are presented with frequency and percentage. Further, considering the complex nature of the study design, survey means of disability weights with 95% confidence intervals (CI) were computed using the VAS scores.

Computation of disability weights (DWs) was done using the formula:


DW=1−VAS/100

Significance tests for comparisons across states, locations (rural and urban), age groups, gender, literacy level, and socio-economic status was done using the analytical statistics. *P*-values below 0.05 were considered statistically significant. Additionally, disability weights derived from this study were compared to those obtained from the global burden of diseases using the Spearman rank correlation.

### Ethics Consideration

The study was approved by the Institutional Ethics Committee of the Indian Institute of Public Health, Bhubaneswar vide IEC no. IIPH/IEC/2017/20. Informed consent was obtained from all participants.

## Results

Socio-demographic profile of the participants interviewed in the study has been presented in Table 2. A total of 2,055 participants were recruited for the study from the two states among which a higher proportion of men from Telangana and a higher proportion of women from Odisha were interviewed. The majority of participants belonged to the age group of 18–34 years, were Hindus, and belonged to the advantaged caste in Odisha as compared to almost half of the participants (47.5%) in Telangana that belonged to the lesser advantaged castes. More than 70% participants were literate, and one-third were homemakers in both the states.

**Table 2:** Socio-demographic profile of the participants.

**Categories**	**Odisha (*N* = 1,013)**	**Telangana (*N* = 1,042)**
**Age (in years)** ***n*** **(%)**
18–34	461 (45.5%)	478 (45.9%)
35–54	400 (39.5%)	469 (45.0%)
55 and above	152 (15.0%)	95 (9.1%)
Mean age (Range)	37.9 (18–80)	36.8 (18–75)
**Sex** ***n*** **(%)**
Male	479 (47.3%)	518 (49.7%)
Female	534 (52.7%)	524 (50.3%)
**Literacy** ***n*** **(%)**
Literate	802 (79.2%)	733 (70.3%)
Illiterate^*^	211 (20.8%)	309 (29.7%)
**Income contribution** ***n*** **(%)**
Contributing	540 (53.3%)	639 (61.3%)
Non-contributing	136 (13.4%)	87 (8.4%)
Homemakers	337 (33.3%)	316 (30.3%)
**Religion** ***n*** **(%)**		
**Hindu**	766 (75.6%)	941 (90.3%)
**Muslim**	47 (4.7%)	74 (7.1%)
**Christian**	199 (19.6%)	23 (2.2%)
**Others** ^#^	1 (0.1%)	4 (0.4%)
Caste^**^n **(%)**
General	453 (44.7%)	171 (16.4%)
Scheduled caste	78 (7.7%)	318 (30.5%)
Scheduled tribe	302 (29.8%)	58 (5.6%)
Other backward class	180 (17.8%)	495 (47.5%)

* ,

** *Definition according to Census and NFHS (National family health survey)*;

# *includes Jains, Buddhists, Sikhs*.

Further as seen in Table 3A, the survey mean disability weights in Odisha and Telangana with urban and rural locations have been shown. We noted that DWs in Odisha ranged from 0.32 (0.30–0.34) for the upper limb fracture due to road traffic accident (least severe) to 0.90 (0.88–0.93) for breast cancer (most severe) among the 14 health states, while, in Telangana, diarrhea was considered least severe [DW = 0.22 (0.19–0.24)] and breast cancer as most severe [DW = 0.85 (0.83–0.88)], similar to Odisha. We also noted that a marked difference in the DWs for alcohol use disorder was perceived as more severe by the communities in Odisha [DW = 0.73 (0.71–0.76)] as compared to Telangana [DW = 0.52 (0.50–0.55)]. Communicable diseases, such as tuberculosis, were considered moderately severe across both the states with almost negligible differences [Odisha: DW = 0.59 (0.57–0.62), Telangana: DW = 0.57 (0.55–0.60)]. Further, two mental disorders, depression and schizophrenia, were included in the list of health states. Depression was valued by the rural participants and was considered more severe in Odisha [DW = 0.63 (0.60–0.63)] and comparatively less severe in Telangana [DW = 0.57 (0.66–0.58)]. However, DWs for schizophrenia did not show any marked difference across the two states.

**Table 3A:** State-wise survey mean disability weights across urban and rural locations.

**Health states**	**Odisha (*****N*** **=** **1,013)**			**Telangana (*****N*** **=** **1,042)**		
	**Urban survey mean**	**Rural survey mean**	**Pooled mean**	**Urban survey mean**	**Rural survey mean**	**Pooled mean**
	**(95% CI)**	**(95% CI)**	**(95% CI)**	**(95% CI)**	**(95% CI)**	**(95% CI)**
Tuberculosis	0.55 (0.54–0.57)	0.63 (0.60–0.67)	0.59 (0.57–0.62)	0.57 (0.54–0.61)	0.56 (0.55–0.59)	0.57 (0.55–0.60)
Diabetes	0.52 (0.49–0.55)	0.56 (0.54–0.58)	0.54 (0.52–0.56)	0.54 (0.52–0.56)	0.56 (0.55–0.57)	0.55 (0.52–0.57)
Diarrhea^*^	0.34 (0.32–0.35)	0.48 (0.43–0.52)	0.40 (0.38–0.43)	0.21 (0.19–0.23)	0.22 (0.20–0.24)	0.22 (0.19–0.24)
Anemia^*^	0.44 (0.42–0.46)	0.63 (0.58–0.68)	0.53 (0.51–0.56)	0.48 (0.45–0.51)	0.44 (0.42–0.47)	0.46 (0.44–0.48)
Breast cancer^*^	0.90 (0.88–0.92)	0.91 (0.89–0.93)	0.90 (0.88–0.93)	0.83 (0.81–0.86)	0.87 (0.86–0.88)	0.85 (0.83–0.88)
Malaria^*^	0.36 (0.34–0.38)	0.45 (0.38–0.54)	0.41 (0.38–0.43)	0.30 (0.28–0.33)	0.30 (0.29–0.31)	0.30 (0.28–0.33)
Asthma^*^	0.57 (0.53–0.61)	0.59 (0.57–0.62)	0.58 (0.56–0.60)	0.50 (0.48–0.51)	0.51 (0.49–0.52)	0.50 (0.48–0.52)
Alcohol use disorder^*^	0.69 (0.66–0.72)	0.78 (0.76–0.80)	0.73 (0.71–0.76)	0.50 (0.48–0.51)	0.55 (0.51–0.59)	0.52 (0.50–0.55)
Fracture^*^	0.33 (0.30–0372)	0.31 (0.26–0.36)	0.32 (0.30–0.34)	0.50 (0.47–0.54)	0.54 (0.51–0.57)	0.52 (0.50–0.55)
Stroke	0.80 (0.78–0.81)	0.84 (0.83–0.85)	0.82 (0.79–0.84)	0.80 (0.78–0.83)	0.81 (0.80–0.82)	0.81 (0.79–0.83)
Oral cancer^*^	0.88 (0.86–0.90)	0.88 (0.87–0.89)	0.88 (0.86–0.90)	0.80 (0.77–0.81)	0.83 (0.80–0.86)	0.81 (0.79–0.84)
Depression	NA^**^	0.63 (0.60–0.67)	0.63 (0.60–0.65)	NA	0.57 (0.55–0.59)	0.57 (0.55–0.58)
Schizophrenia	0.64 (0.60–0.67)	NA	0.64 (0.62–0.66)	0.66 (0.62–0.71)	NA	0.66 (0.64–0.69)
Osteoarthritis^*^	0.32 (0.28–0.36)	0.43 (0.37–0.50)	0.38 (0.35–0.40)	0.48 (0.46–0.50)	0.49 (0.46–0.53)	0.49 (0.46–0.51)

* *The pooled mean disability weights for Odisha and Telangana were found to be statistically significant (p <0.05) in these health states*.

** *Depression was valuated only by rural inhabitants whereas Schizophrenia was valuated only by urban inhabitants; 95% CI; 95% confidence interval*.

Further as seen in Table 3B, all the health states were perceived to be less severe in urban areas than the rural areas. For instance, DW for anemia was 0.46(0.45–0.53) in urban locations whereas 0.53(0.45–0.53) in rural locations because city people may be taking into account better access to healthcare services.

**Table 3B:** Mean disability weights for different health states across Location (urban/rural).

**Health states**	**Urban survey** **mean- (95% CI)**	**Rural survey** **mean- (95% CI)**	***p*-value**
Tuberculosis^*^	0.56 (0.56–0.59)	0.59 (0.56–0.59)	0.0000
Diabetes^*^	0.52 (0.53–0.55)	0.56 (0.53–0.55)	0.0000
Diarrhea^*^	0.27 (0.25–0.35)	0.34 (0.25–0.35)	0.0000
Anemia^*^	0.46 (0.45–0.53)	0.53 (0.45–0.53)	0.0000
Breast cancer^*^	0.86 (0.86–0.89)	0.88 (0.86–0.89)	0.0038
Malaria^*^	0.33 (0.31–0.39)	0.35 (0.31–0.39)	0.0000
Asthma^*^	0.52 (0.51–0.56)	0.54 (0.51–0.56)	0.0853
Alcohol use disorder^*^	0.59 (0.57–0.67)	0.65 (0.57–0.67)	0.0002
Fracture	0.42 (0.37–0.47)	0.42 (0.37–0.47)	0.3460
Stroke^*^	0.80 (0.80–0.82)	0.82 (0.80–0.82)	0.0001
Oral cancer^*^	0.84 (0.82–0.86)	0.85 (0.82–0.86)	0.2100
Depression	NA	0.65 (0.62–0.68)	-
Schizophrenia	0.65 (0.62–0.67)	NA	-
Osteoarthritis^*^	0.40 (0.40–0.45)	0.46 (0.40–0.45)	0.0000

* *The pooled mean disability weights were found to be statistically significant (p <0.05) in these health states*.

*Depression was valuated only by rural inhabitants whereas Schizophrenia was valuated only by urban inhabitants; 95% CI; 95% confidence interval*.

A Spearman-rank order correlation test was done to compare the DWs obtained from our study and the GBD 2015 study, as seen in Table 4. We observed that when the community weights were compared to GBD 2015 weights, the correlation was found to be low (ρ = 0.104). However, the Spearman-rank order correlations between the two states were high as well as statistically significant (ρ = 0.82, *p* = 0.0002), indicating a similar rank ordering.

**Table 4:** Comparison of the community disability weights with GBD 2015 weights.

**Group 1**	**Group 2**	**Spearman correlation (ρ)**	***p*-value**
Community	GBD	0.104	0.721
Odisha	Telangana	0.823	0.0002^*^

* *p <0.005*.

## Discussion

Results from our pioneering community-based Health State Valuation (HSV) method could establish that through the use of simple and easy-to-use valuation methods DWs for health states can be estimated with high levels of overall concordance across diverse communities, representing to a large extent the heterogeneity of the Indian population. Therefore, DWs can be used to estimate national and subnational disease burden(s) in the Indian context. By using art and science through the use of visual analog scale method and the individual images of functional status describing varying levels of each dimension of a health state, we believe our study has added value in assessing disability weights among populations with mostly lower levels of education.

To prioritize health research and interventions, donors and countries need to have concrete and reliable data in terms of the burden of diseases. The 1990 GBD study was an important step toward DW calculation and burden estimation (17). However, in later GBD studies, until almost a decade ago, the perspective of professional healthcare providers was assumed to be representative of the society's preferences with regard to resource allocations in health care. Gradually, it was realized that health as well as healthcare are greatly influenced by an individual's perception, education, culture, environment, and life experiences, across communities, states, countries, and regions (18). Hence, the weights obtained from GBD studies were not regarded universally representative and garnered criticism across the world (19). Moreover, for allocation of resources or designing interventions intended for the marginalized population, DWs needed to be accurate and representative of the community. Therefore, to address this gap in developing countries, a study by Mahapatra et al. in 1999 was conducted in a village in Andhra Pradesh to obtain India-specific disability weights (11). Though the study was able to capture location-specific DWs, the cultural diversity of our country, with the changing disease patterns and the rising burden of NCDs, urgently required an update on the local DWs. People belonging to different social status, education level, and health state have different perceptions regarding health (18). Hence, our study is pilot in nature and thus an initial attempt to assess community disability weights for selected health states that varied in terms of severity across different locations and covered a varied population from different sections of the society, including the urban slums and rural areas.

Through the use of simple and easy-to-use valuation methods, we were able to successfully achieve high levels of overall concordance across diverse communities that represented a heterogeneous mix of the population. Similar to the GBD 2010 disability weights measurement study, our study aspired to quantify health loss as opposed to welfare loss (19). The extra-welfarist approach was used in our study, which considers health as the descriptive entity of the people (11). This approach allowed for the use of rating scales as the visual analog scale for the measurement of disease severity and establishing DWs. Previous studies have shown clear cultural differences in the ways people perceive health problems and how such problems affect their lives. This was endorsed by Üstün et al., who found significant differences in the ranking of health states between 14 countries (20). Furthermore, the findings from Jelsma et al. and Baltussen et al. suggest that the effect of cultural differences on health perceptions should be reflected in the DWs as well, and hence there is a need to develop socio-culturally contextualized weights (7).

In our study, disability weights obtained for different health states were more or less universal, in the sense of being uniform or similar across locations, states, and cultures. Card sort and visual analog scale methods were thus chosen rather than the cognitively demanding methods (15, 16) that usually include specialists. The health state with most the variability in terms of DWs was alcohol use disorder (AUD) with a DW of 0.73 in Odisha as compared to a lower disability weight of 0.52 in Telangana, suggesting that AUD was perceived as less severe in Telangana. Similarly, upper limb fracture due to a road traffic accident was considered as more severe in Telangana (DW = 0.52) than in Odisha (DW = 0.32). Probable reasons that affected the perception could be awareness and availability of treatment in both the states that vary greatly. This divergence reflects how the local context and culture shape disability perception of communities. Further, for health states affecting physical conditions such as quadriplegia due to an episode of stroke or osteoarthritis, the DWs were more uniform than the mental health states across states and locations (18). We also note that although there were significant differences between health states, the factor regarding the duration (acute vs. chronic) should also be noted, as health states with shorter duration were most often than not scored with a higher disability weight. Additionally, due to the small sample size of nonslum participants (130 out of 2,055), our study has presented a limitation for exploring differences between slum and nonslum populations. We also highlight that a disadvantage of using a less cognitively challenging tool such as VAS gives higher values than that from choice-based valuation methods.

Our study provided the evidence based on disability weights derived from community settings for comparison with the GBD disability weights since the valuation of health states was highly correlated across the two states in the study. Furthermore, our pilot disability weight study covered relevant health states that are required for updating the burden of disease study in the country and can be used for the next GBD as well. However, the present methodological pilot attempted to capture DWs of two neighboring Indian states. Additional research, especially of qualitative nature, is needed to gain greater insight into the effects of cultural differences on disability weights, particularly across the country in varied settings. Further, studies should intend to include the entire spectrum from noninfectious, non,-contagious conditions to highly infectious, noncommunicable, and nationally relevant health states that would be a great value addition to the national disease burden estimates and health policies.

## Data Availability Statement

The raw data supporting the conclusions of this article will be made available by the authors, without undue reservation.

## Ethics Statement

The study was approved by the Institutional Ethics Committee of the Indian Institute of Public Health, Bhubaneswar vide IEC no. IIPH/IEC/2017/20. Informed consent was obtained from all participants. The patients/participants provided their written informed consent to participate in this study.

## Author Contributions

EL, SA, CS, and RR were involved in the conception and design. EL and SA were involved in drafting of paper. EL and AD were involved in the revision of the manuscript. LN and GM were involved in critical analysis of the paper. All authors have approved the final version of the article submitted.

## Funding

This study was funded by the Indian Council of Medical Research Grant Number 58/13/NCD-BOD/Health-V/2016-NCD-II.

## Conflict of Interest

PD and SA was employed by IQVIA and Care India. The remaining authors declare that the research was conducted in the absence of any commercial or financial relationships that could be construed as a potential conflict of interest.

## Publisher's Note

All claims expressed in this article are solely those of the authors and do not necessarily represent those of their affiliated organizations, or those of the publisher, the editors and the reviewers. Any product that may be evaluated in this article, or claim that may be made by its manufacturer, is not guaranteed or endorsed by the publisher.
