# Supplementary material for: Disability Weights Estimates From India in 2018: Measurements From Community Members From Two Distinct States of India
Source: Front Public Health. 2022 Mar 22;10:752311. doi: 10.3389/fpubh.2022.752311 (PMC8980316; doi:10.3389/fpubh.2022.752311)

VISUAL DESCRIPTION OF HEALTH STATE VALUES AMONG FEMALES

1. Anemia

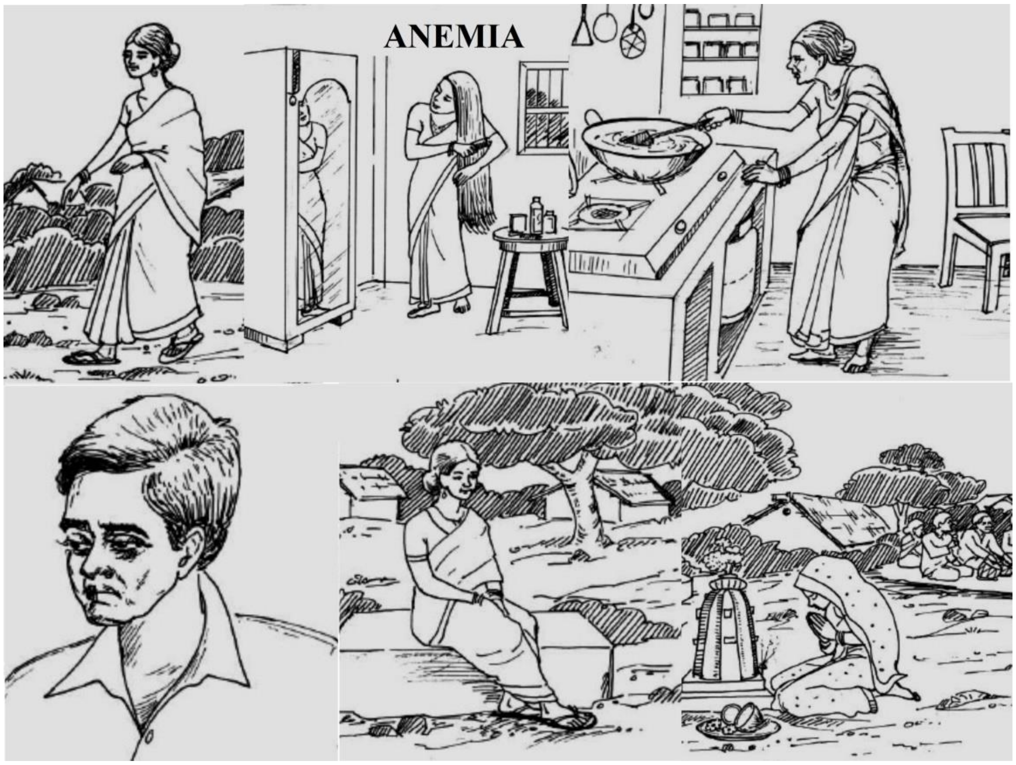

2. Asthma

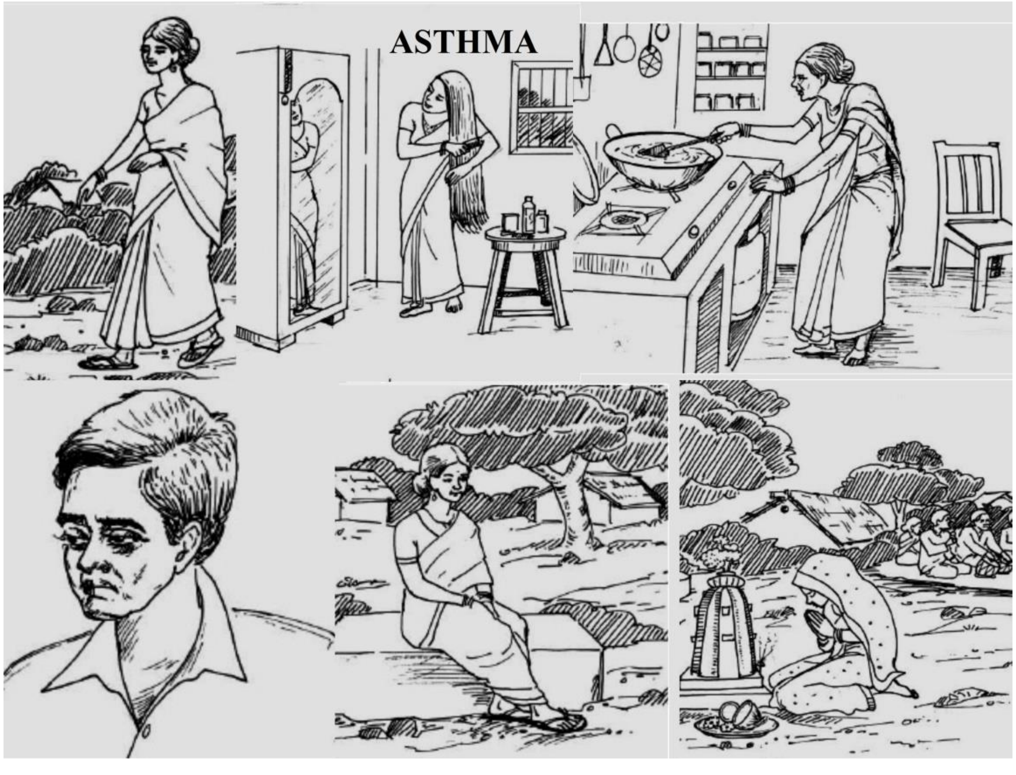

3. **Breast Cancer**

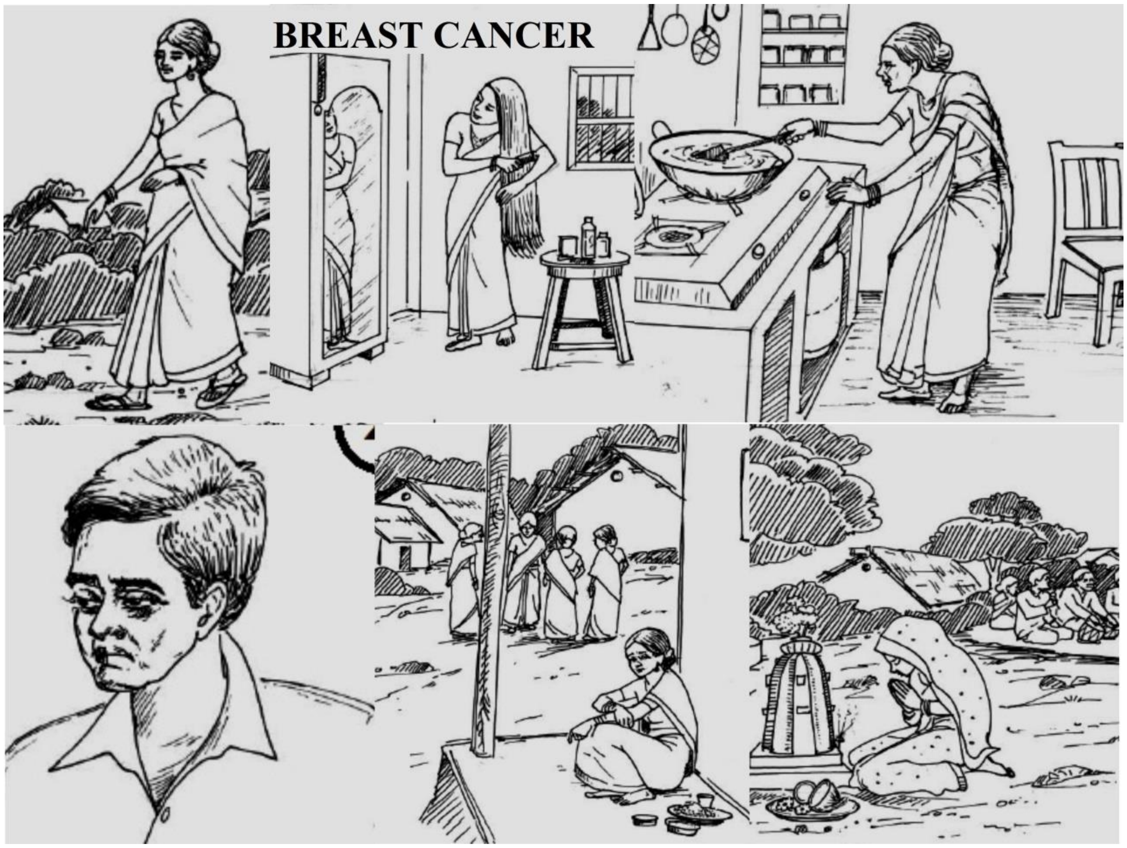

4. **Depression**

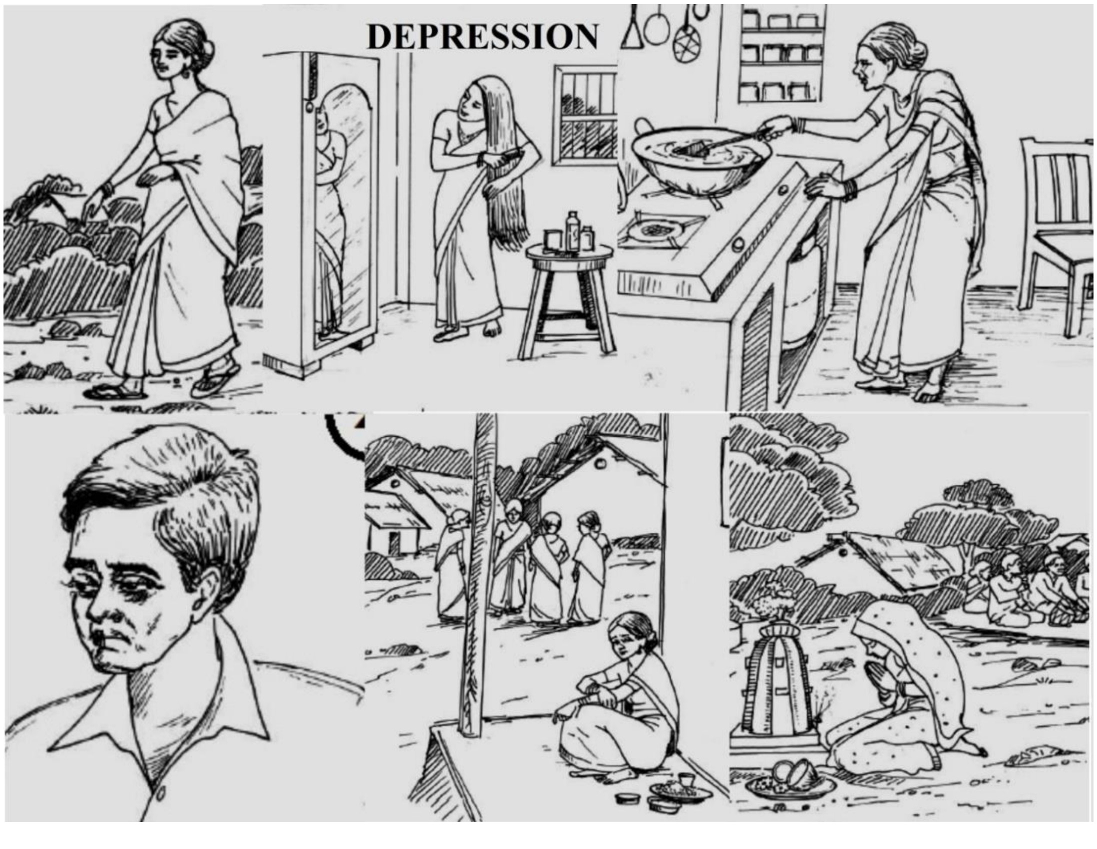

5. Diabetes

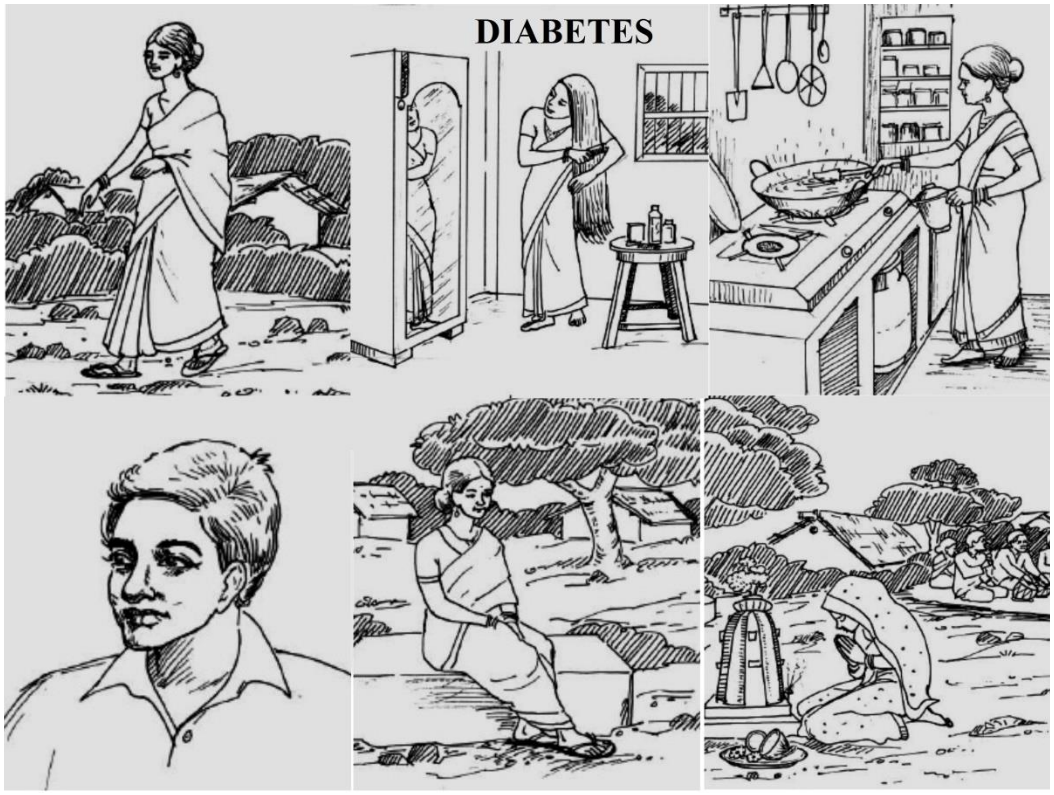

6. Diarrhoea

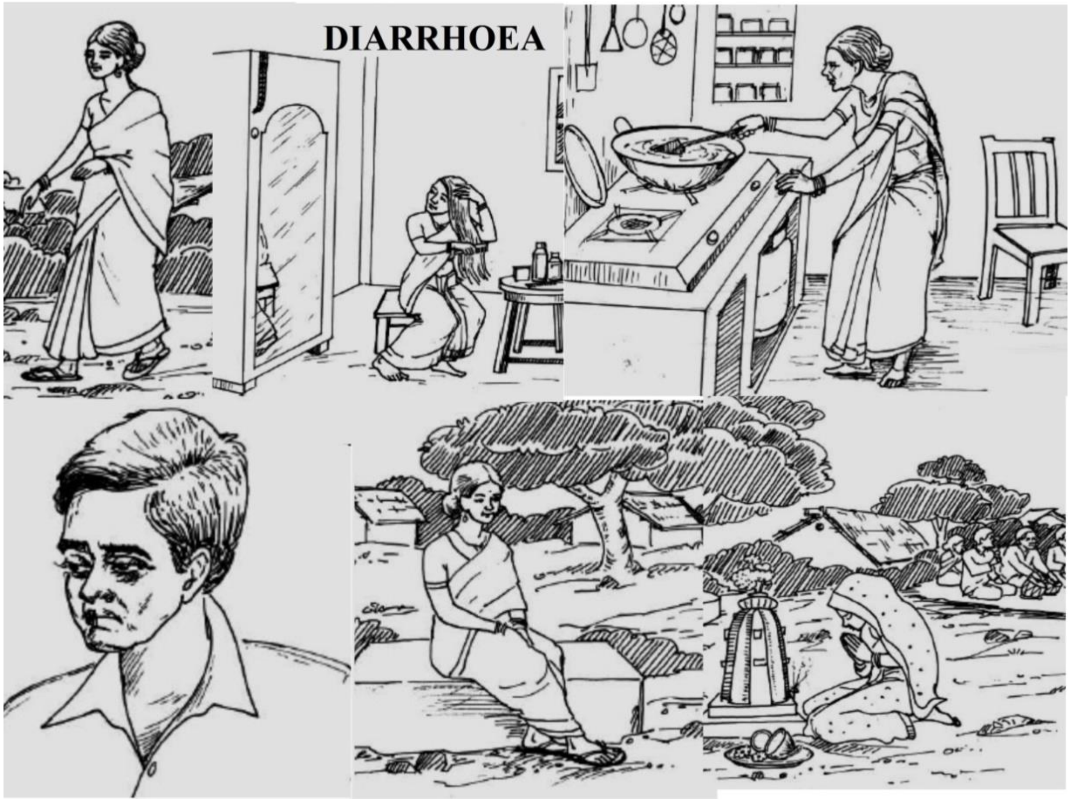

7. Malaria

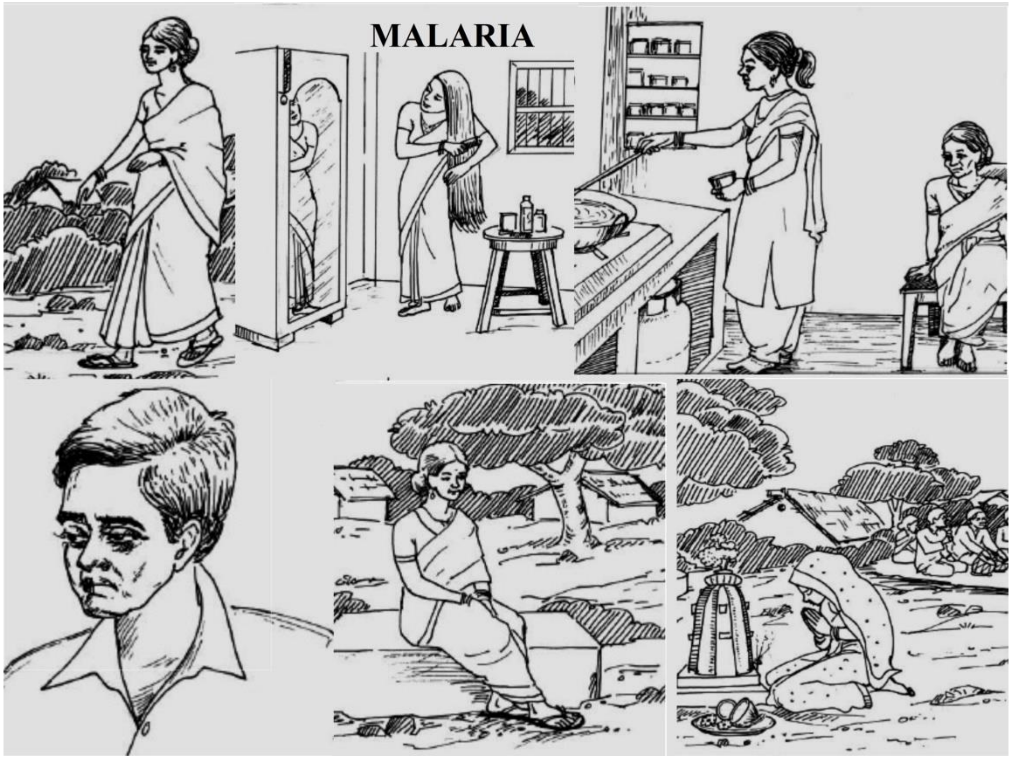

8. Osteoarthritis

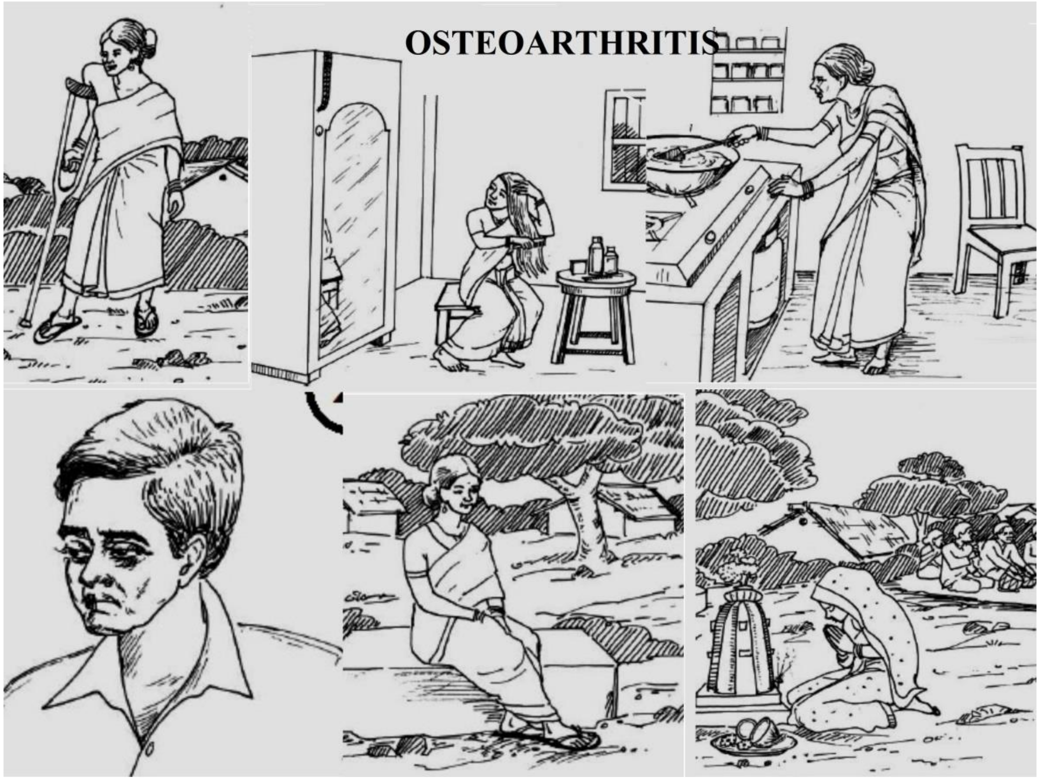

9. Schizophrenia

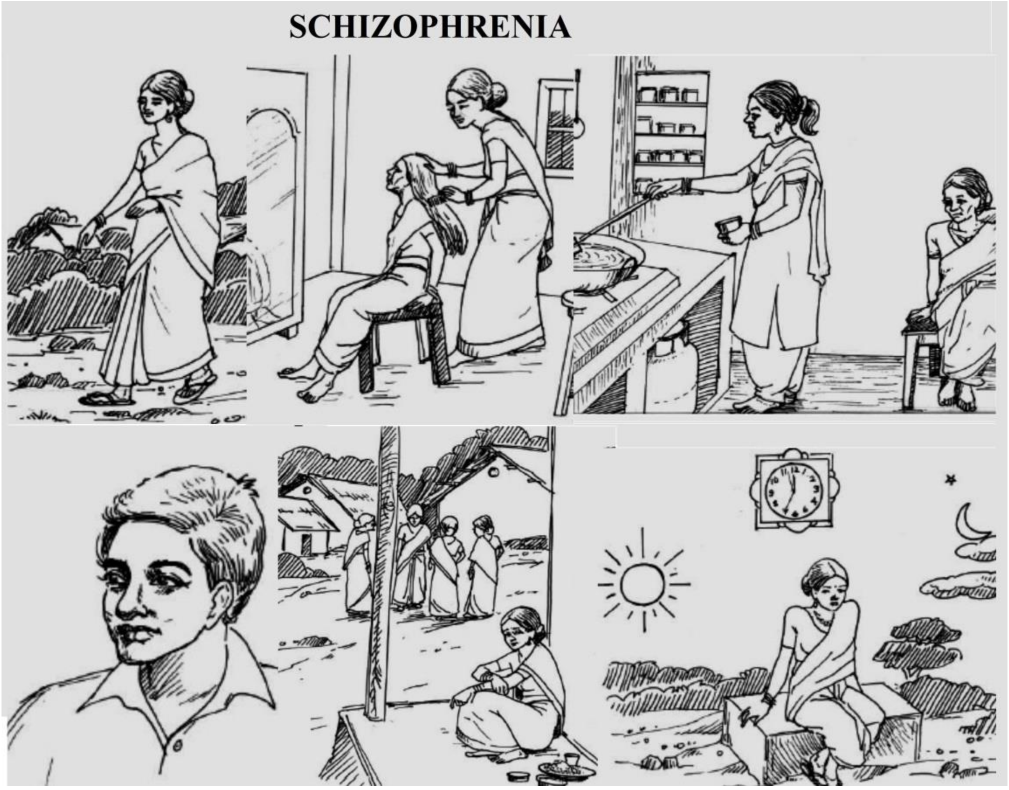

10. Stroke

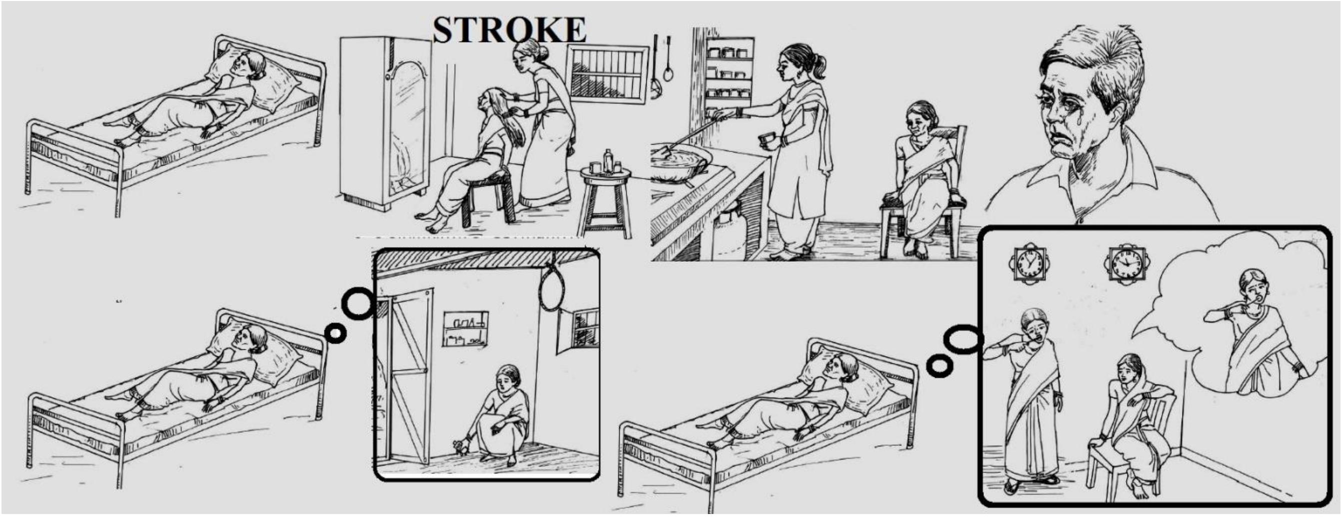

11. Tuberculosis

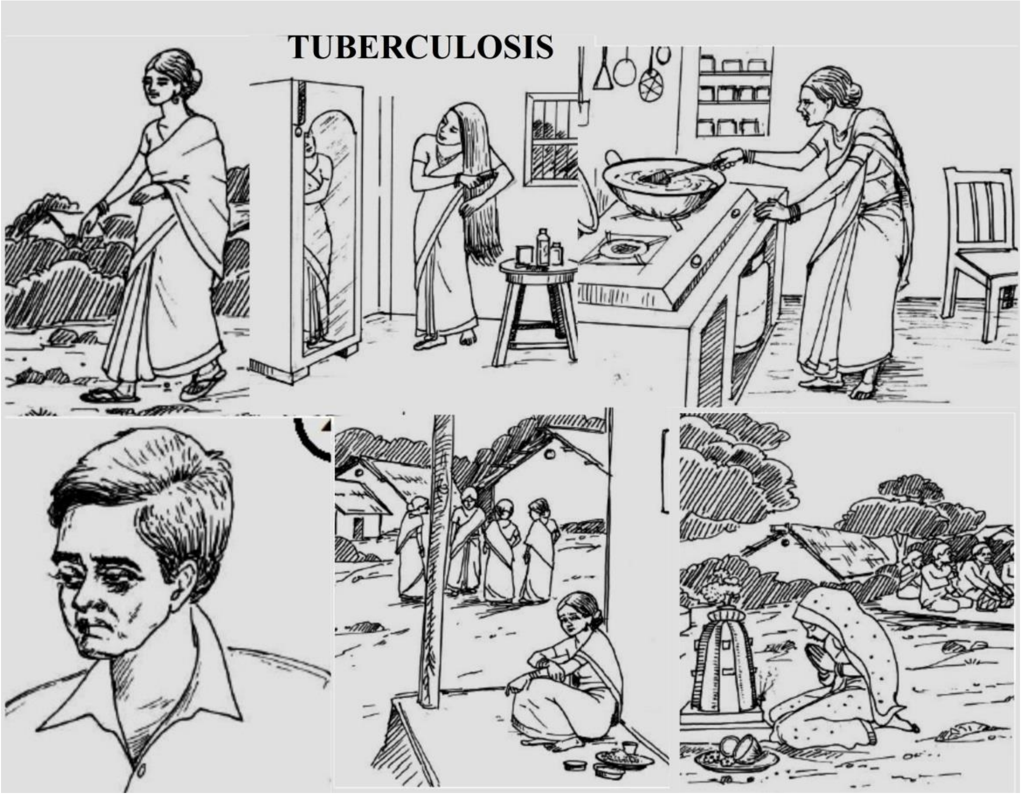

12. Upper limb fracture

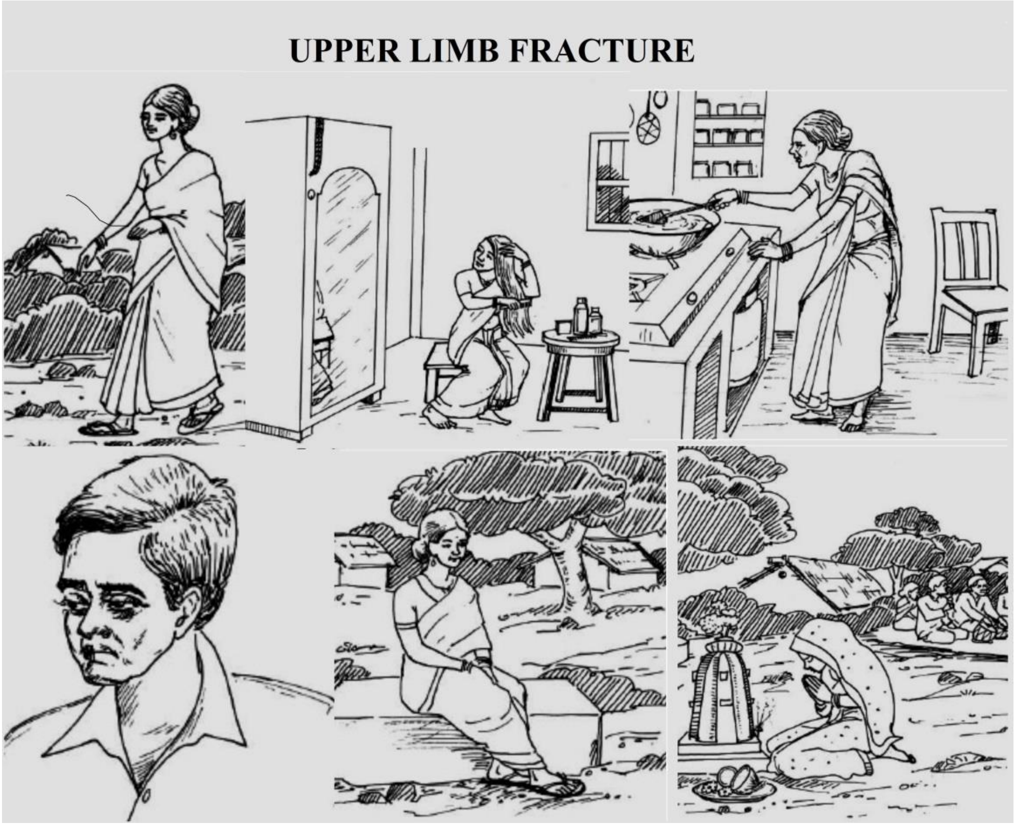

Supplement: Supplementary file 1 [file Data_Sheet_1.PDF]
