# Supplementary material for: Disability Weights Estimates From India in 2018: Measurements From Community Members From Two Distinct States of India
Source: Front Public Health. 2022 Mar 22;10:752311. doi: 10.3389/fpubh.2022.752311 (PMC8980316; doi:10.3389/fpubh.2022.752311)

VISUAL DESCRIPTION OF HEALTH STATE VALUES AMONG MALES

1. **Alcohol Use Disorder**

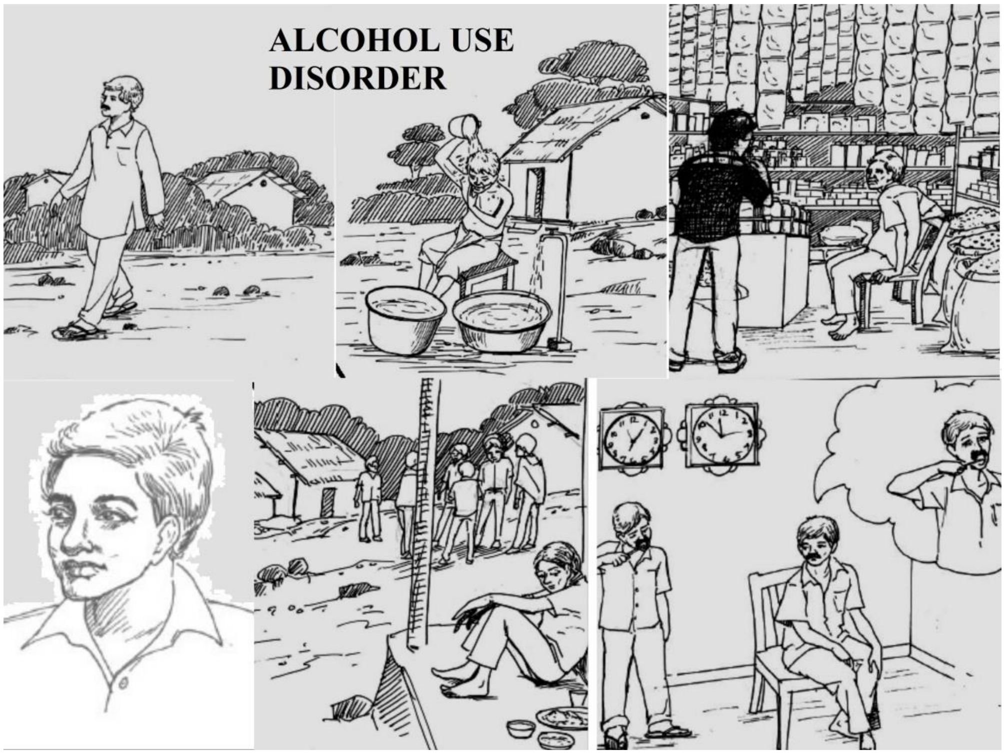

2. **Asthma**

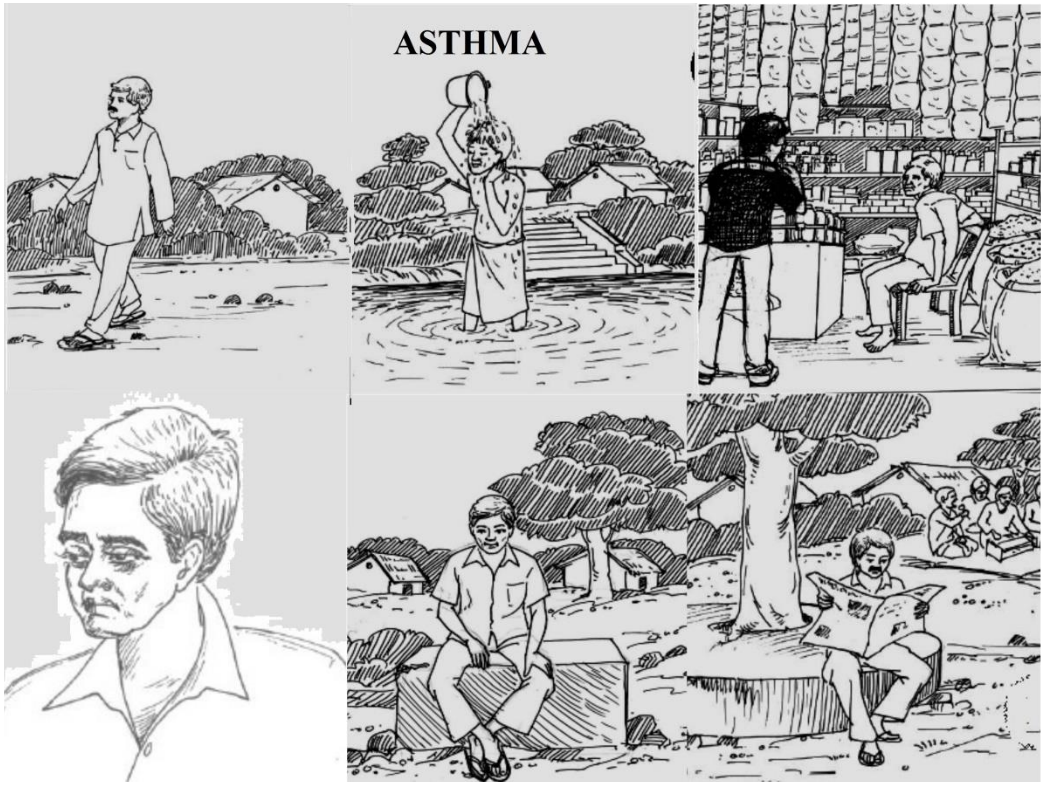

3. Diabetes

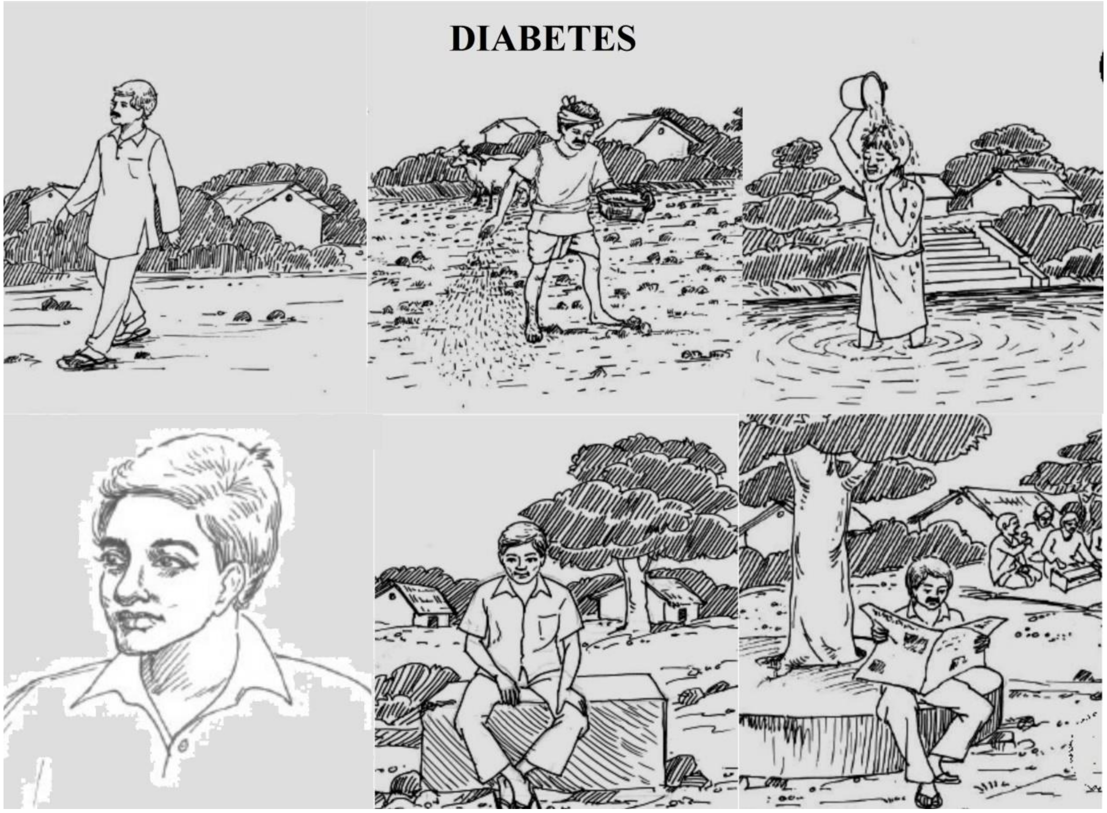

4. Depression

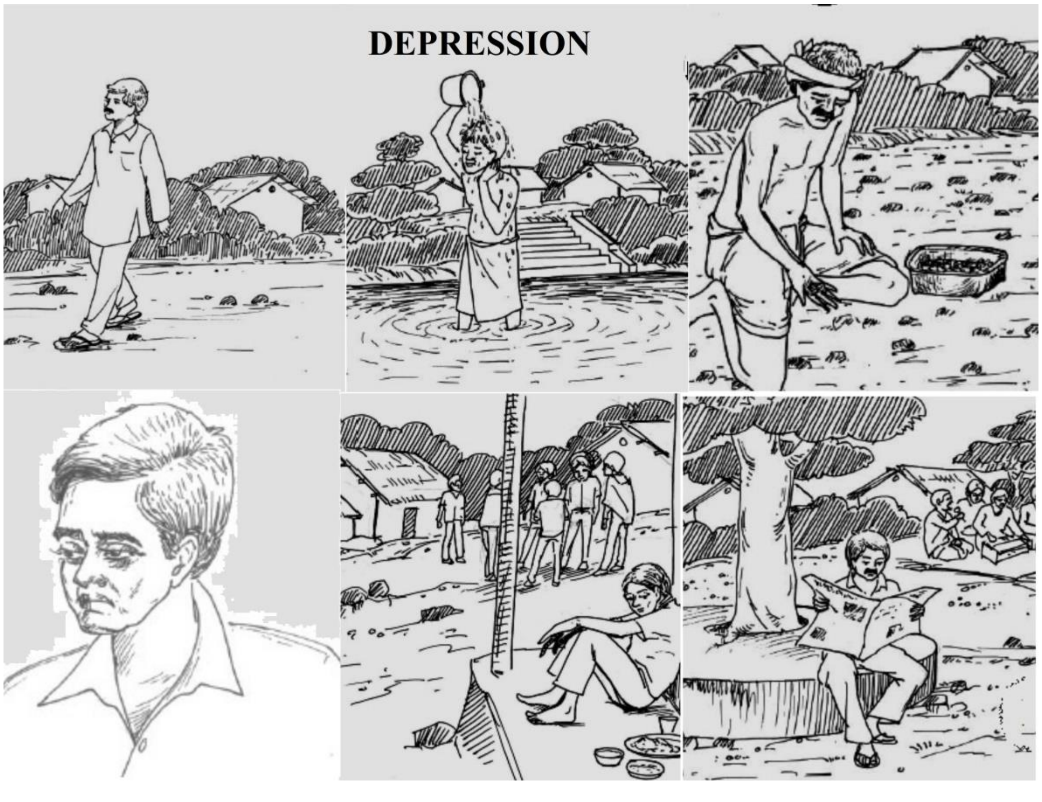

5. Diarrhoea

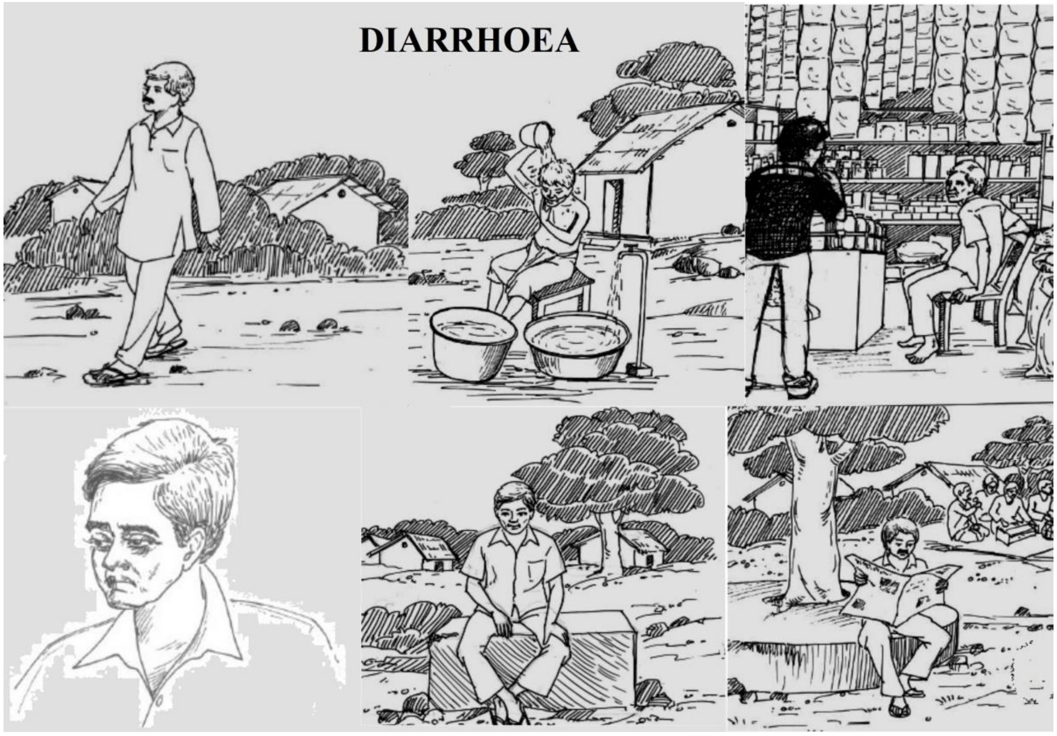

6. Malaria

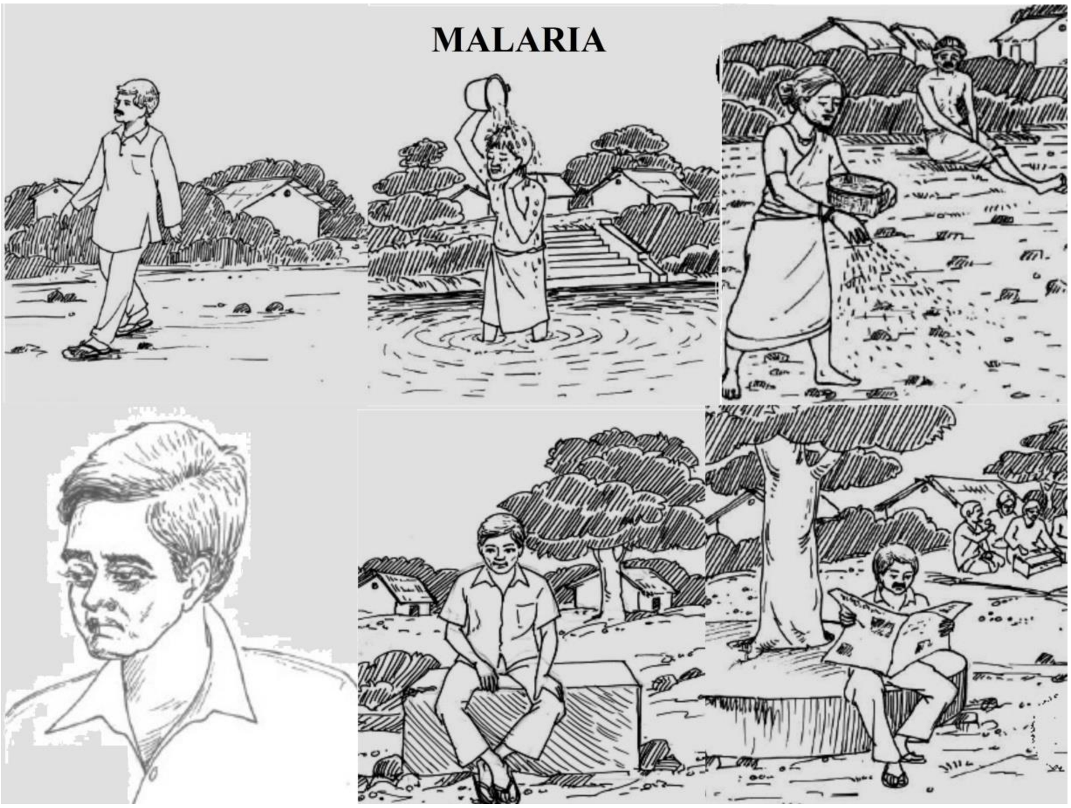

7. Oral Cancer

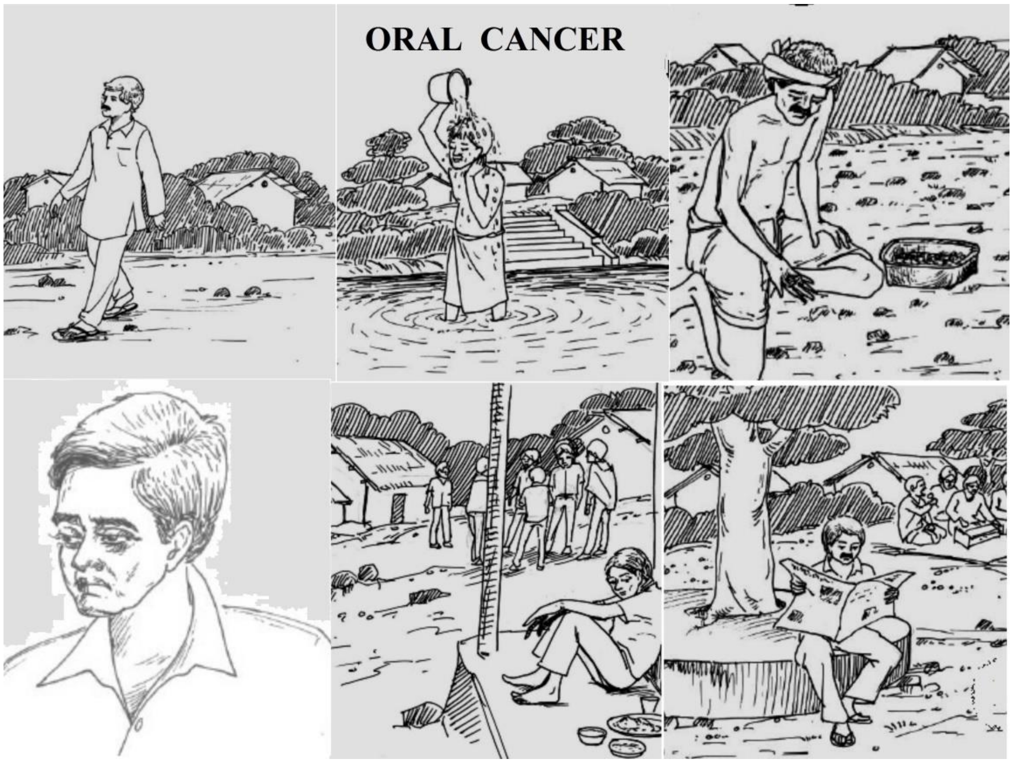

8. Osteoarthritis

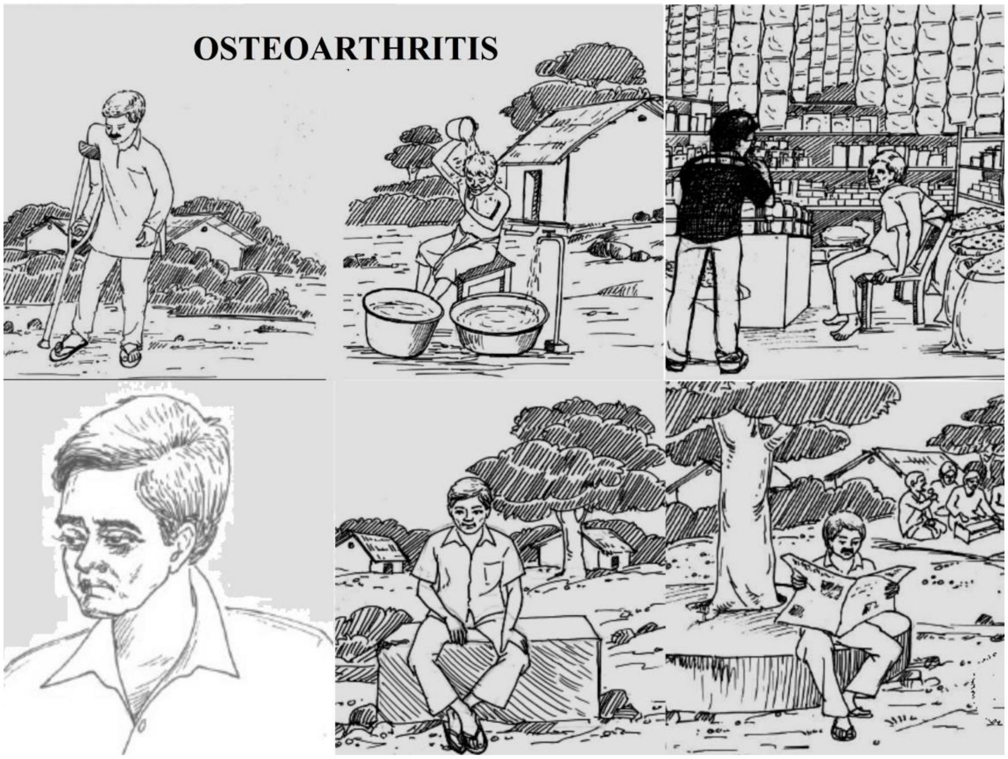

9. Schizophrenia

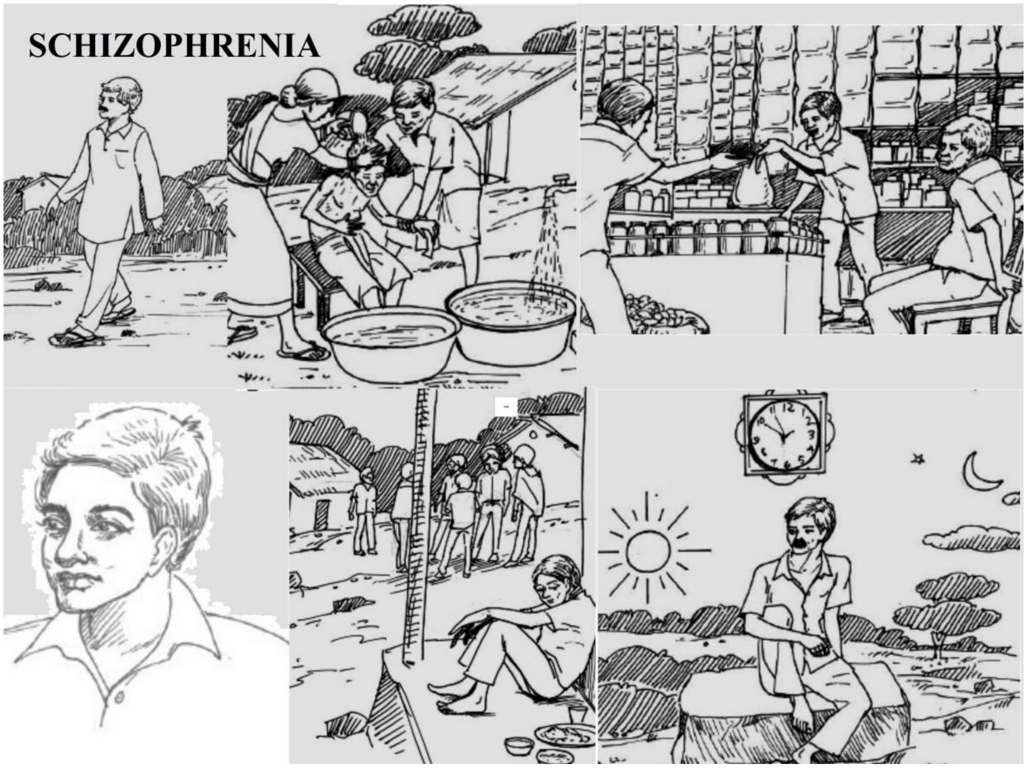

10. Stroke

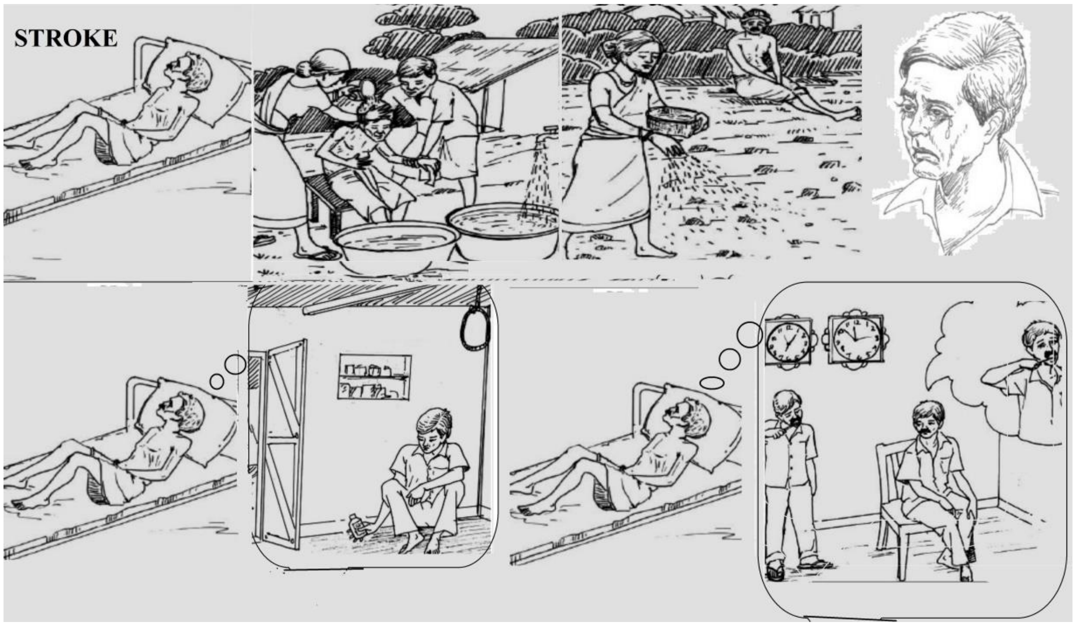

11. Upper Limb Fracture

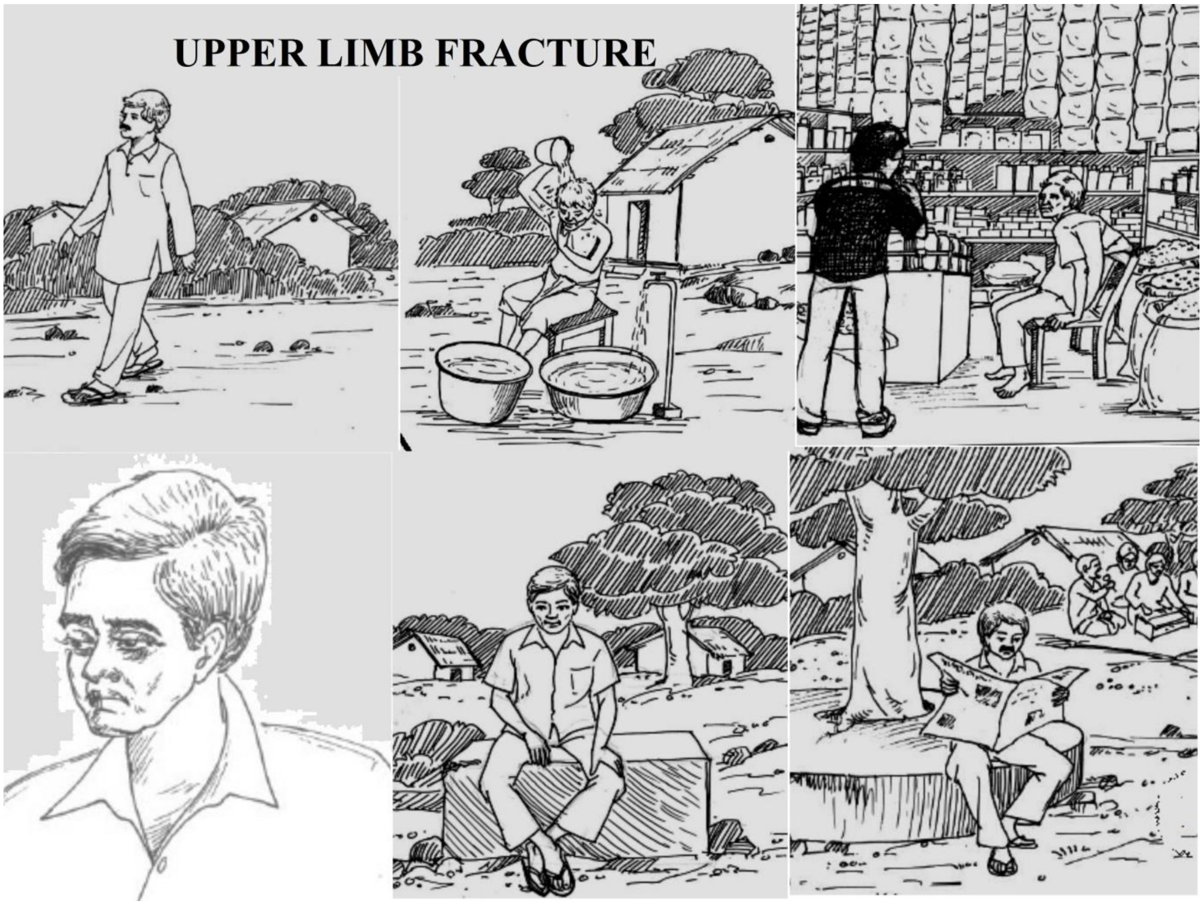

Supplement: Supplementary file 2 [file Data_Sheet_2.PDF]
